# All-cause and cause-specific mortality amongst individuals with substance misuse: A population-based linked data study in Wales

**DOI:** 10.23889/ijpds.v9i5.2798

**Published:** 2024-09-18

**Authors:** Grace Bailey, Ian Farr, Hywel Evans, Saira Ahmed, Columbus Ohaeri, Lauren O'Gorman, Ryan Phillips, Olabambo Oluwasuji, Josh Dixon, Joanne Maimaris, Josie Smith

**Affiliations:** 1 Swansea University; 2 Public Health Wales; 3 Welsh Government

## Objective

Substance misuse has a detrimental impact on an individual’s health and the wider society, as well as elevated mortality. Here, we analysed the association between substance misuse events across several healthcare service providers and risk of mortality.

## Approach

In this retrospective population-based cohort study, we identified substance misuse amongst a cohort in Wales, aged ≥10 years old between 2010 and 2019 using the Secure Anonymised Linkage (SAIL) Databank. Cox regression and Kaplan-Meier survival curves were used to calculate risk for all-cause and cause-specific mortality.

## Results

During the study period 16,229 (9.7%) individuals with a substance misuse event died. A high proportion of these individuals died prematurely (<75 years, 66.7%), and nearly half were from the most deprived areas at time of death (46.8%). Alongside alcoholic liver disease and alcohol hepatic failure, cardiovascular and respiratory diseases were the most common underlying causes of death. Those in receipt of specialist substance misuse treatment for alcohol-use were at greatest risk of suicide (hazard ratio: 1.83) and drug-related deaths (hazard ratio: 1.98). Risk of suicide was also higher for those with a drug-use health event recorded during a hospital admission (hazard ratio: 1.89).

## Conclusions

Substance misuse was associated with elevated mortality risk, in particular, amongst those with substance misuse recorded during inpatient hospital admissions. These findings highlight the need for early interventions such as increased screening and monitoring of substance use to prevent premature mortality.

## Implications

Our results provide a clear indication of service needs to support planning and provisioning of health services.

